# Three dimensional shape comparison of flexible proteins using the local-diameter descriptor

**DOI:** 10.1186/1472-6807-9-29

**Published:** 2009-05-12

**Authors:** Yi Fang, Yu-Shen Liu, Karthik Ramani

**Affiliations:** 1 School of Mechanical Engineering, Purdue University, West Lafayette, IN 47907, USA; 2 School of Electrical Computer Engineering (by courtesy), Purdue University, West Lafayette, IN 47907, USA

## Abstract

**Background:**

Techniques for inferring the functions of the protein by comparing their shape similarity have been receiving a lot of attention. Proteins are functional units and their shape flexibility occupies an essential role in various biological processes. Several shape descriptors have demonstrated the capability of protein shape comparison by treating them as rigid bodies. But this may give rise to an incorrect comparison of flexible protein shapes.

**Results:**

We introduce an efficient approach for comparing flexible protein shapes by adapting a *local diameter *(LD) *descriptor*. The LD descriptor, developed recently to handle skeleton based shape deformations [1], is adapted in this work to capture the invariant properties of shape deformations caused by the motion of the protein backbone. Every sampled point on the protein surface is assigned a value measuring the diameter of the 3D shape in the neighborhood of that point. The LD descriptor is built in the form of a one dimensional histogram from the distribution of the diameter values. The histogram based shape representation reduces the shape comparison problem of the flexible protein to a simple distance calculation between 1D feature vectors. Experimental results indicate how the LD descriptor accurately treats the protein shape deformation. In addition, we use the LD descriptor for protein shape retrieval and compare it to the effectiveness of conventional shape descriptors. A sensitivity-specificity plot shows that the LD descriptor performs much better than the conventional shape descriptors in terms of consistency over a family of proteins and discernibility across families of different proteins.

**Conclusion:**

Our study provides an effective technique for comparing the shape of flexible proteins. The experimental results demonstrate the insensitivity of the LD descriptor to protein shape deformation. The proposed method will be potentially useful for molecule retrieval with similar shapes and rapid structure retrieval for proteins. The demos and supplemental materials are available on .

## Background

The importance of protein molecule shape has been recognized in many structural biological applications such as computer aided molecular design, drug discovery, and protein structure retrieval [2-4]. The similarity comparison of protein molecule shape plays a central role in understanding of protein functions [4-8] of molecular systems, and leads to an alternative way to discover the specific nature of proteins efficiently besides the similarity measure from the alignment of sequence [9,10] and secondary structure [11-16]. The underlying assumption is that the geometrically similar molecules are likely to share similar properties. The shape based methods could be made independently from chemical structural knowledge. The attractive advantage of shape based methods make protein comparison alignment-free and therefore very efficient. Some methods show their effectiveness for comparing proteins using the their surface shape [4-7,17], but they cannot handle the deformation of the flexible protein well because proteins are treated as rigid bodies. This intrinsic weakness leads to significant comparison errors when flexible proteins in different conformation states are compared to each other.

Our work concentrates on a shape-based method for the deformation invariant shape representation using the *local diameter *(LD) *descriptor *for flexible protein comparison. The proposed method will mainly be used in any type of molecular shape comparison (MSC) based applications [2,3,18,19], for example, virtual screening of compounds for similar shapes for drug discovery, and protein structure retrieval based on shape similarity [4].

### Shape comparison

The importance of a comparison of the shape of 3D objects' shape is increasing in the areas of computer vision, robotics, molecular biology, and others. The shape is usually expressed by a descriptor, which is a feature vector capturing some essence of a given shape [20-22]. A shape descriptor generally carries a number of desirable properties: transformation invariant, succinctness for representation, low computation expense, expressiveness of shape, etc. The concise form of the shape descriptor is of great benefit in shape comparison. Instead of alignment and superposition, a shape descriptor allows for the efficient shape comparison since only a simple calculation of some distance between feature vectors is required [23].

Additionally, the shape descriptor can be stored as an index in a database of shapes to enable real time query and retrieval. Protein shape analysis has been developing as an indispensable way to overcome the challenge of reaching the goal of disclosing the unknown functions of proteins. In the next part we review several recent works related to a protein shape.

The global protein shape descriptor proposed in [24] introduces a method to compare and classify proteins based on their topological properties. In [25], spherical harmonic expansion was applied to compare the protein binding pocket and ligand. A 3D shape is decomposed via spherical harmonics, and followed by computation of the distance in the coefficient space to evaluate the similarity among proteins. Petros [7] applied the spherical trace transform to a protein shape to produce a rotation invariant shape descriptor. Ingolf [6] successfully brought the moment invariants to the comparison of protein binding sites. In [26,27] the authors introduced a technique to generate compact descriptors of the shapes of molecules and of macromolecular receptor sites. The ray-tracing technique is used to explore the volume enclosed by a ligand molecule, or the volume exterior to the active site of a protein. The shape descriptor is expressed as a histogram of the ray segment lengths obtained from the ray tracing within the volume of the molecule. Ballester et al. developed a shape comparison method named USR for screening a huge database compound to find out the similar molecular shapes [2,3]. Furthermore, Lee [4] used a 3D Zernike descriptor to represent the global surface shape. The protein surface is compactly expressed as a series expansion of three-dimensional functions. The authors used the global surface shape similarity based technique for fast protein tertiary structure retrieval. By using this simple descriptor for indexing the database, it only takes seconds to search against a few thousand proteins.

### Local diameter descriptor

Proteins undergo structural changes and shape deformation upon protein-protein or protein-ligand interaction [28-30]. A particular conformation state carries out a specific function of the protein. Beginning with the native conformation, the protein usually adapts its structure to changes in its surrounding environment [29,30]. It is of importance to represent shapes of different protein conformations in an invariant form. Invariancy is associated with the shape descriptor not being effected by the deformation of the protein shape. None of the descriptors introduced above can support deformation in shape for comparison and matching. This is because those shape descriptors change significantly with deformation as well as topological changes.

Local diameter (LD) scalar function is defined on the boundary surface of a 3D shape. LD has been recently developed for a pose-oblivious shape descriptor in [1]. Furthermore, the authors demonstrated that the local diameter descriptor is invariant of skeleton based shape deformation in [31]. We applied the LD descriptor to capture the invariant properties of the shape deformation due to the motion of the protein backbone. The proposed novel approach includes three steps for characterizing the protein shape. First, a certain number of points is sampled from the protein surface. Then, every sampled point is assigned a value measuring the local diameter of the 3D shape in the neighborhood of that point. Lastly, a shape descriptor in the form of a 1D vector is built from the distribution of the local diameters. The LD descriptor captures the invariant essentials of the shape deformation and is expressed in a 1D vector. By using the LD descriptor, not only could we represent the shapes of flexible proteins consistently, but also we could characterize the protein shape independent of its atom coordinates and structural details. This approach is very computationally efficient since it is free of structural alignment and superposition.

## Methods

In this section, we first provide a definition of the LD descriptor and then the procedure to compute the LD descriptor to represent the protein shape. The procedure starts with the description of the approach to extract the boundary points, followed by a comparison of sampling techniques. Next we present LD descriptor calculation algorithms, and finally, we explain the similarity measurement methods for shape descriptors.

### Definitions

The LD descriptor is a type of statistic based descriptor. It starts with sampling boundary points, measures the shape diameters on every sample point, and generates the one-dimensional histogram. In [1], the local diameter is defined as the distance from one surface point to the antipodal surface point using the inward-normal direction. The authors also proved that the shape diameter is an approximation of twice the shape radius [32]. Shape radius measures the distance from a surface point to the object medial axis (skeleton). The shape radius is demonstrated to be invariant to skeleton based shape deformation.

However, the existing approaches suffer from the complexity of computing the skeletons [32]. Local diameter is an alternative way to simplify and approximate the shape radius without any computation of the object's skeleton. Therefore, while preserving the properties of shape radius, the definition of local diameter is invariant to rigid body transformation (rotation, translation, and uniform scale), articulated deformations, and skeleton based movements [31]. We could simulate the protein hinge-bending motion as one type of skeleton-based movement [33-35]. Therefore, the LD descriptor is reliable for capturing the invariancy associated with the shape deformation caused by the protein backbone's motions.

### Algorithms

The local diameter is defined by a statistical measure of the diameters in a cone around the direction opposite to the normal of the point. The detailed algorithms of how to compute the LD descriptor are summarized as follows:

1. Extract the boundary from the whole volumetric model and sample boundary points,

2. Measure the local diameter for every sample point, and

3. Build the LD descriptor and score the similarity between descriptors.

#### Boundary points

##### Detection of boundary points

The volumetric representation, popularly used in biological research fields [4,5,36], is used to represent protein shape in this paper. A volumetric model is composed of a set of cubic units named voxels, which is the 3D counterpart of the 2D pixel of an image. A voxel is centered at an integral grid point and assigned a numeric value representing some measurement, for example, the density [37,38]. The volumetric model is built in three steps as follows. First, we compute the Connolly surface (triangle mesh) of the molecule using the MSROLL program [39]. Second, we place the triangle mesh in a 3D cubic grid of *n*^3 ^(e.g. n = 65) compactly. Third, each lattice point is assigned either 1 or 0; 1 for point inside the surface and 0 for outside. The inside point is denoted as object point and the outside point is denoted as background point. For each lattice point, there is a set of 26 neighbor points. An object point lies on the boundary if at least one of its 26 neighbors is a background point.

##### Subset of boundary points

**Figure 1:**
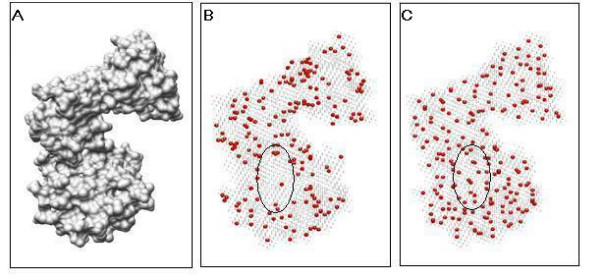
**Comparison of clustering sampling and random sampling techniques and effect**. (A) The protein surface. (B) The red points depicted are the random sample points. (C) The red points depicted are the clustering sample points. Note that in figure(B) the ellipse marks a surface region where the sampled points distribute very sparsely, while the same surface region includes evenly distributed sampled points by using the clustering sample technique. The sample size is set at 300 in the figures.

The full boundary point set is too large to compute shape signature effectively. To save storage and computation costs, we carefully choose a subset but preserve the characteristics of the shape. To have statistically valid sample data, the sample density/size should be carefully determined since the more samples we take, the more accurately and precisely we can reconstruct the shape distribution; however, at the same time, a large set of sample data increases the storage and expense of shape signature computation exponentially. In addition, the sample method is also taken into account to yield a representative data set. We compared random sampling and clustering sampling methods. With random sampling, every member in the boundary point set has an equal chance of inclusion in the sample set. The points picked out by the random sampling method cannot yield an informative sample set. In this paper, we utilize the K-means [40] algorithm to implement a clustering sampling approach. The K-means algorithm divides the boundary points into groups based on the distances among them. Statistically, points from the same cluster have similar properties, and the cluster center can be selected to represent the whole set of points expressively. In the experiment, the sample size is set as the number of groups, and the point nearest to the cluster center is chosen as the final sample point. Figure 1 shows a comparison of sample points for a protein model using random and clustering methods. We find that the clustering-based sample method can approximate the shape better than random sampling given the sample size. The points sampled randomly look unevenly distributed on the surface and then lose the local shape information. Note that the elliptical highlighted region in Figure 1(B) includes almost no points, while the same region in Figure 1(C) has evenly distributed sampled points.

#### Local diameter calculation

The algorithm for finding the local diameter for a boundary point has the following steps.

1. Estimate the normal of the boundary point,

2. Define a virtual cone using this point as a vertex, centered around the opposite direction of the point's normal with a proper opening angle, and shoot a certain number of rays inside the cone to the opposite side, and

3. Intersect a ray with the opposite boundary side and record the distance from the point to the antipodal intersection point, and

4. Sort the distances and use the median as the local diameter.

**Figure 2:**
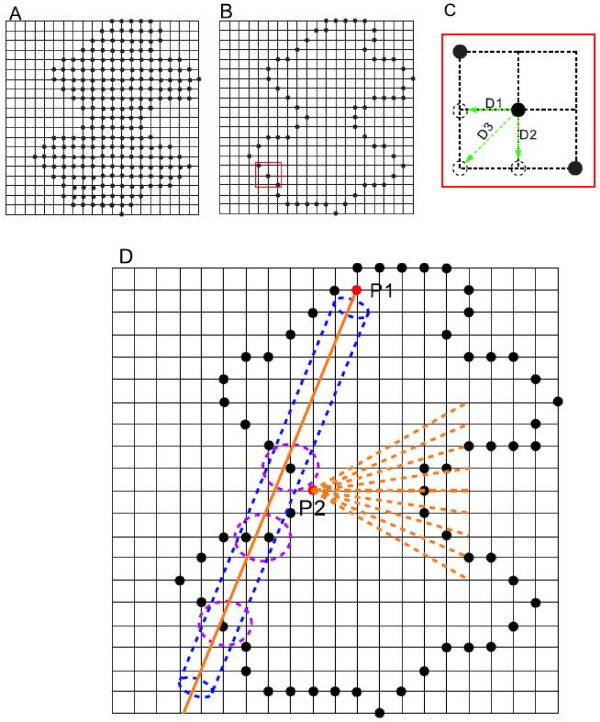
**Two-dimensional illustration of the scheme for the algorithm**. (A) Volumetric representation of protein. The solid dots denote the set of protein data placed on the uniform grids. The grids without solid dots are outside of the protein. (B) The boundary contour of the protein. The region highlighted by a red square is used for the illusion of normal calculation. (C) The figure zooms in the highlighted region in (B) to illustrate the calculation of the normal. The center point is one of the boundary points and three open dots are defined as its visible points. The three green arrows denote the visible direction vectors. The normal is the vector summation of *D*_1_, *D*_2 _and *D*_3_. (D) The visualization of ray intersection. A virtual cone (colored orange) with *P*_2 _as vertices is shown in the figure. A ray (colored orange) shot from point *P*_1 _and the corresponding virtual cylinder (colored blue) around the ray are shown. There are three circles (colored purple) describing the different clusters of intersection points respectively.

The above steps are applied on all sample points. Figure 2(D) is a two-dimensional representation, showing the visualization of the use of ray, cone, and cylinder. Details of the algorithm for the normal estimation (step two) and intersection calculation (step three) are provided below.

##### Normal estimation

The normal of the boundary point is unknown in the volumetric model. We introduce an efficient technique based on visibility to estimate the normal of the boundary points [5]. The basic idea of the normal estimation is illustrated in Figure 2(C). The visible direction for a boundary point is defined as the vector linking it to one of the outside points in its neighbor. The green arrows in Figure 2(C) denote three visible vectors of the center point. The normal of a point is measured by the vector sum of all the visible directions. In the case of Figure 2(C), the normal for the center point is the vector sum of *D*1, *D*2, and *D*3. The calculation of the normal performs the same procedure in the 2D and 3D cases except that the number of neighbors of one point is 8 in the 2D case while it is 26 in the 3D case.

##### Ray intersection

Intersecting a ray with different objects such as a sphere can be directly checked if the equations describing the objects are known. Since we use a volumetric model, the only known information is the coordinate of the point. Hence, we introduced a robust algorithm [41], called LPSI (Line and Point Sets Intersecting), for resolving the intersection problem. This algorithm is fast and robust and obtains high accuracy without requiring a reconstruction of the underlying surface from the point cloud. This method consists of four steps:

1. Consider a virtual cylinder around the ray shot from the cone vertices,

2. Detect whether an intersection has occurred between that cylinder and boundary points and collect the inclusion points, and

3. Cluster the inclusion points, and

4. The nearest cluster is picked up as the final intersection point.

Note that the reader can find the algorithm details in [41]. We provide a 2D illustration to demonstrate the framework for the algorithm. See Figure 2(D). The protein surface is represented as a solid dot contour; the black dots represent the boundary points. In Figure 2(D), an orange ray is shot from red point *P*1, and a blue virtual cylinder is around the ray. We can see there are three clusters of intersection points, highlighted by three purple circles. The number of points included in the three clusters is one, two, and two respectively. The intersection algorithm picks up the nearest cluster as the final intersection point of the ray with the opposite boundary side.

#### Similarity measurement of shape descriptor

The last step is to express the values of the local diameters in the form of a one-dimensional histogram vector. After obtaining the local diameter of every boundary point, we compute the distribution of those values and build the histogram of values using 128 bins to define the descriptor. In order to compare the LD descriptor with popular shape descriptors, we introduce a distance descriptor, called Euclidean distance (ED) [20]. The ED descriptor is formed by three steps: 1) sampling points from the shape surface, 2) computing the Euclidean distance between the pairwise sampled points, and 3) computing the distribution of distance values to build the histogram given the number of bins. Note that the distribution is estimated by counting the number of observations of distance values falling into a specific range adjusted by the number of bins. The histogram built from the distribution indicates the number of distance values per unit bin.

In order to quantize the measurement of the shape similarity, we need to define an appropriate scoring function for the distance metric in some vector space. As mentioned above, the shape descriptor is a histogram based 1D vector and is constructed by dividing the distance range from the maximum to minimum measurement into 128 equal sized ranges and counting the number of observations that fall into different ranges. Therefore, each descriptor is a 128 length long 1D vector which can be expressed mathematically as *I *= (*I*_1_, *I*_2_,..., *I*_*k*_) *k *= 128. There have been a number of standard ways of comparing two vectors as described in [20]. The popular standards include *L*1 norm, *L*2 norm, *λ*^2 ^measure, and Bhattacharyya distance. In fact, the experimental results tell us that using different metrics slightly affects the comparison and retrieval results. Although we tested different types of metrics for shape descriptors, we have found that *L*1, and *L*2 norms are simple and give better results. The equations for *L*1, known as Manhattan distance, and *L*2 norm, known as Euclidean distance, are given below:

(1)

(2)

where *N*_*bin *_is the number of bins of histogram, and *I*_*A *_and *I*_*B *_denote shape descriptors of the two proteins, A and B, respectively.

## Results

We have implemented the LD descriptor and assessed its performance from the experimental results. The experimental comparison of the LD descriptor and existing structure based and shape-based methods will be studied in future work while applying the LD descriptor in different applications. The algorithms presented in the paper are implemented on a Pentium D 3.2 GHz computer with 1 G RAM running Windows XP. The proteins for the experiments have been chosen from the Database of Macromolecular Movement (MolMovDB) [42], which categorizes conformational changes of protein and allows the user to animate and visualize a particular motion through the Morph server. This database has been used in predicting protein structures and hinge predictor [43,44]. There are two critical parameters, the opening angle of the cone and the number of rays in the cone that have to be carefully chosen in the experiments. The importance of cone angle has been discussed in [1,31]. A small cone angle would make the LD descriptor too sensitive to local features and would lead to poor discrimination between different objects as well. On the other hand, a large opening angle would bring extra noise and errors due to the rays intersecting to unrelated parts of the object [31]. The number of rays indicates the sampling density for rays shooting from the vertex within the cone. The number of rays would not affect much the performance of the LD descriptor. The authors in [1] tested the effect of various parameter settings and found that the opening angle of 120° and 50 rays are reasonable settings. In the experiments, we followed the setting in [1] choosing 120° and 50 rays. Here, we are not focused on a detailed analysis of the biological significance of the proteins, but the robust performance of the shape descriptor and we analyze the possible reasons causing incorrect comparison.

### Visualization of Local Diameters

**Figure 3:**
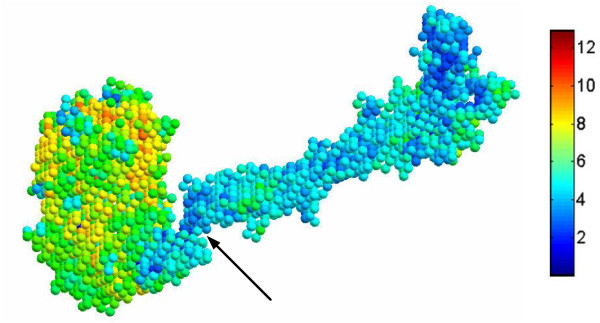
**Illustration of local diameter for surface points**. Different colors represent different values of the local diameter. The colorbar on the right side indicates the range of values.

Figure 3 shows a protein (PDB: 1QCRE) with color regions on the surface. The conformational deformations of this protein can be viewed from the Morph Server. In the figure it is shown that the protein consists of a domain and a long helix chain. We can see in the motion movies on the web-server that the domain rotates about the hinge (pointed by an arrow in the figure) to different extents. Surface points are colored differently according to their local diameter values. The color bar on the right side indicates that a red color means the large diameter and the blue color means a small diameter. The figure shows that the points from the domain are predominately yellow and green while the points from the helix chain are predominately blue. This supports the notion that the domains have much larger diameters than those of the helix chain. Furthermore, because the protein surface is usually undulated, this causes some surface points from the domain to have a small local diameter. We can find that those very small protrusions located on the surface of the domain are blue colored, indicating that those points have a low value of local diameter.

To summarize this section, the LD descriptor can measure the local diameter very well. The body of the domain usually has large diameters.

### Shape Deformation Invariance

**Figure 4:**
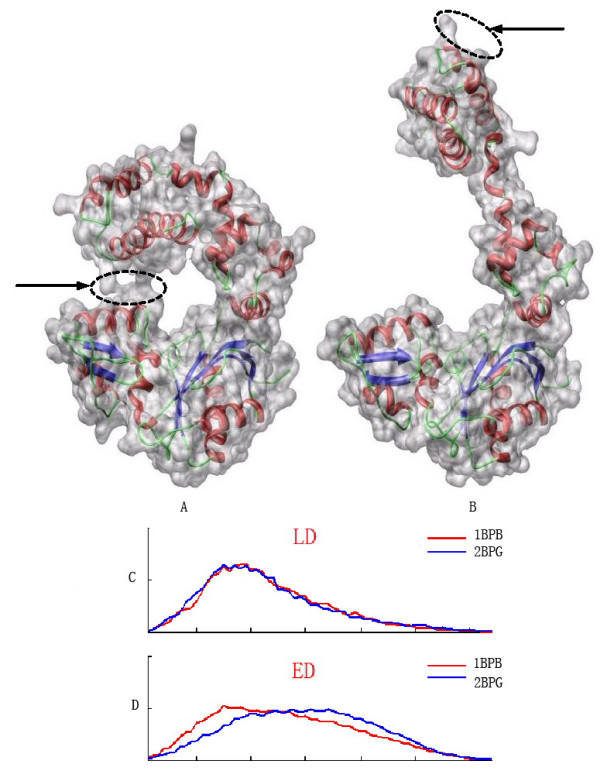
**LD descriptor is compared to the Euclidean distance (ED) descriptor**. The protein in (A) transforms into the protein in (B) by bending the top portion of the protein. The arrow points to the touching regions between the top portion and the bottom rigid part, which means that the topology changes during this deformation. (C) shows the LD descriptor. (D) shows the ED descriptor. The red and blue color are used to distinguish two different proteins (PDB: 2BPG (left) and PDB: 1BPB (right)). The LD and ED descriptors in (C) and (D) are two histogram based vectors. Here each histogram is constructed by dividing the distance range from the maximum to minimum measurement into 128 equal size ranges and counting the number of observations that fall into different ranges. The horizontal axis of the histogram is labelled with the range of distance measurement, and the vertical axis of the histogram is the number of observations that falls into the corresponding range. Note that if *I*_*A *_represents the descriptor (red plot) and *I*_*B *_represents the descriptor (blue plot), the similarity can be measured using equations (1) or (2). The LD descriptors are consistent with two proteins and are not sensitive to shape deformation and topological changes. In contrast, the ED shape descriptor is strongly sensitive to those changes.

In order to show the potential of the LD descriptor as shape deformation invariant, we choose some proteins that have conformational changes for test purposes. We tested some complex shape deformations (see Figure 4) to demonstrate the capability of the LD descriptor for its insensitivity to the protein shape deformation.

Two illustrative examples of proteins (PDB: 1BPB and 2BPG) are shown in the top part of Figure 4. The two conformations have a large shape change and even the shape topology is changed due to a small contact (highlighted by an ellipse in the figure) between the top portion with the bottom domain. The LD descriptor is shown in Figure 4(C). The plot shows that the histograms for the two shapes match well, indicating that the shape descriptor is effective enough to be invariant to the complex shape deformation even with topology changes. In contrast, the ED shape descriptor method fails to represent the shape deformation invariantly as shown in Figure 4(D) since there is a large deviation between the two shape descriptors.

### Database Retrieval Performance

#### Benchmark Dataset

**Figure 5:**
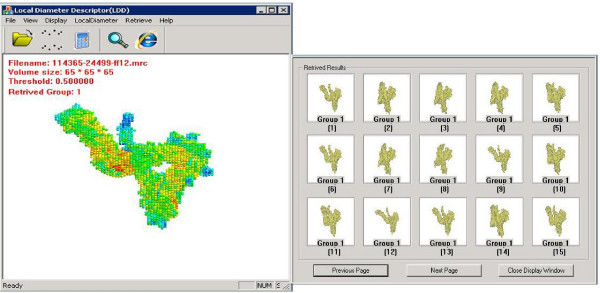
**Protein Shape Search Engine**. The left side shows the file information, such as filename, and group ID, of the query protein and the right side shows the retrieval results. The group ID is indicated underneath the retrieval result. Note that we can see that the first 15 retrieval results match the query protein correctly.

To further investigate the effectiveness, we test the LD descriptor on a benchmark dataset of proteins extracted from MolMovDB, which consists of 2,695 proteins classified into 214 groups. The Database of Macromolecular Movements  is a collection of diverse data pertaining to flexibility in proteins. Proteins from the same group basically have similar elements of secondary structures but there might be some conformational changes to make spatial orientation different. The hinge bending movement is a major type of motion which leads to the deformation in protein shape. We also developed a search engine (see Figure 5) with which the user can easily visualize the retrieval results and evaluate the retrieval performance. The retrieval results are displayed using images in a dialog box with a group ID label directly underneath every image (see Figure 5). The order of the results sequence is determined by the similarity score between shape descriptors of the query and retrieval.

Before searching, we pre-calculated all shape descriptors of queries in the database and stored them as index files. Therefore, every protein is transformed to a compact 1D vector (e.g. 128 numbers). With the new form of representation, proteins can be retrieved extremely fast since only the distance between 1D vectors needs to be compared. If the query protein is already transformed into a shape signature, it only takes seconds to finish a query retrieval process. Meanwhile, if the query protein is not pre-calculated, the search engine would first build the shape descriptor and search against the database.

#### Specificity Sensitivity Analysis

Specificity and sensitivity are widely used standards to assess the performance in retrieving similar protein structures [4]. The definitions are given as follows:

(3)

(4)

where TP, the number of true positives, is the number of proteins included in the group that are the same as the query protein correctly retrieved in the search; FN, the number of false negatives, is the number of proteins that are included in the group the same as the query protein but missed in the search; FP, the number of false positives, is the number of proteins that are included in a different group from the query protein but inaccurately retrieved in the search. Thus, the denominator in Eq. 3 is the total number of all members in a group, and the denominator in Eq. 4 is the retrieval size.

**Figure 6:**
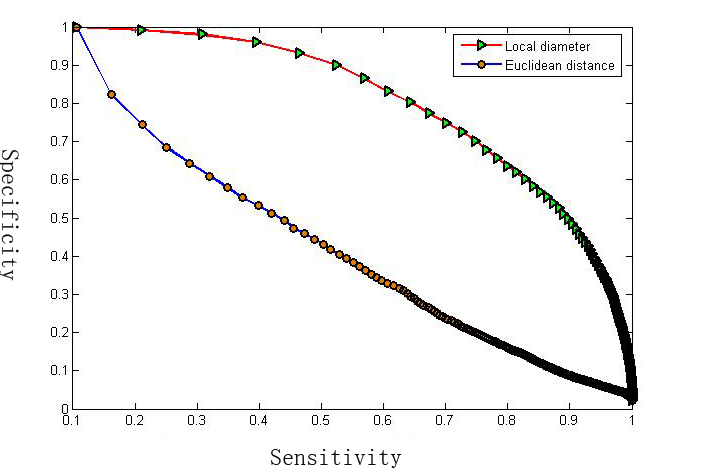
**The sensitivity and the specificity curve**. The retrieval performance of the LD descriptor is evaluated and compared to the ED descriptor. Note that the LD descriptor performs much better than the ED descriptor because its sensitivity and the specificity curve is much closer to tend to the horizontal line at specificity = 1.0.

In the experiment, we first score the similarity between each protein and the whole set of proteins, and then rank them according to the score. The sensitivity and the specificity for every query protein is then recorded. We average all of the records to give the final sensitivity and the specificity. Figure 6 shows the specificity and sensitivity curve of the benchmark performance. The red plot with green triangle marked is the sensitivity-specificity curve for the LD descriptor, and the blue plot with solid dot marked is the curve for the ED shape descriptor [20]. A perfect search retrieves all relevant objects consistently at each sensitivity level, generating a horizontal line at specificity = 1.0. However, practically, the specificity decreases with the increasing sensitivity. The closer the curve tends to the horizontal line at specificity = 1.0, the better the retrieval method. Therefore, we can see from the plots that the LD descriptor performs much better than the ED shape descriptor since the specificity-sensitivity plot for the ED method decreases too fast.

## Discussion

### Analysis of experimental results

We found there are a few cases where the local diameter descriptor cannot express the shape deformations of the shape protein appropriately. Here we list potential reasons causing the inaccuracy. First, the volumetric data is a low resolution record of protein shape. Some details of protein surface are missed during the voxelization process, which ultimately causes the improper representation. Second, the sampling of boundary points would cause the loss of some shape information. Third, the LD descriptor deals well with the shape deformation caused by the motion of the protein backbone. However, in some cases, the hinge or loop is elongated to a large extent, which harms the performance of the descriptor. Furthermore, we found that a few proteins of highly similar shape are assigned to different groups in the Molmovdb database. Our method likely fails to distinguish the proteins from those groups. For instance, there are two groups in the Molmovdb database, which include proteins functioning as ovotransferrin and lactoferrin, respectively. Our method fails to differentiate between these two groups due to the high similarity of the protein shape.

### Potential Applications

As the size of molecular databases increase exponentially, the development of the efficient screening methods has been receiving a lot of attention. We are working on two potential applications of the LD descriptor including the molecular shape similarity search for drug discovery and protein structure retrieval based on the shape surface similarity. There are two aspects of advantages for the LD descriptor in those applications. First, the LD descriptor can handle the flexible molecular shape comparison. Second, the LD descriptor is able to search against a large database rapidly.

#### Molecular shape comparison based drug design

One of the foundations for the rational drug design is the use of shape similarity identified compounds are expected to be active against a given target [2,3,27]. The virtual screening of compounds based shape matching has been recently developed for the drug discovery process [3,18]. The underlying assumption is that the molecular shape has been widely acknowledged as a critical factor for biological activity and geometrical resemblance of molecular is closely associated with the functional similarity [2,3,27]. The recent two works are reviewed as follows. The authors in [27] proposed their shape signature method and applied it to the Tripos fragment database and the NCI database (113,331 compounds) under two different metrics. Ballester et al. [2,3] introduced a ultrafast shape recognition method to finish a inter-database compounds search for similar molecular shapes. The databases used in their experiments include the Vendor Database (2,433,493 commercially available compounds) and an independent benchmark from DrugBank.

The existing shape based techniques first pre-compute a shape signature of the compounds in a large database, and a list of similar shape molecules can be ranked based on some distance metrics with a given lead molecule. In future work we will apply the the LD descriptor to some large and diverse molecular databases for the drug discovery. The LD shape descriptor could provide the alternative to existing molecular shape descriptor with a potentially better performance in terms of searching by matching the 3D conformation of molecules.

#### Hybrid scheme for protein structure retrieval

Fast protein structural retrieval techniques are necessary to deal with the increasing number of known protein structural data. Over the past years, many structural comparison methods have been proposed to solve the structure retrieval problem [11-16]. The widely used structural alignment methods, such as DALI [15] and CE [12], have been proposed to identify the defined best alignment. The structural alignment generally produces a superposition of corresponding atom pairs and the RMSD distance is calculated as the similarity metric. A structural alignment, FATCAT [11], is recently proposed for the flexible structure alignment. In addition, Liu [45] presented a new algorithm based on least median of squares to achieve a flexible structure alignment. The reader can refer [46] for comprehensive review of protein structure alignment methods. Generally, before comparing a pair of structures, an optimal sequence alignment, which has been shown to be NP-hard problem [47], should be first conducted to provide the corresponding residues. Therefore, the structural alignment methods generally have critical weak points in terms of computational complexity although they are able to provide good structure retrieval results.

The LD descriptor is capable of real time retrieving because it is an alignment-free technique, and the descriptor vector could be pre-computed and stored as an index on a hard disk. In the future work we will develop a hybrid scheme which includes both the structure alignment and shape based methods. The LD descriptor could work as a rapid primary filter to obtain the initial candidates and followed with an option to combine structure alignment based methods to refine the initial results. The structural comparison is only applied to the highly ranked protein candidates to reduce the time of a whole retrieval process. With this hybrid scheme, we can balance well between the searching time and the accuracy of structure retrieval results.

#### Limitations

**Figure 7:**
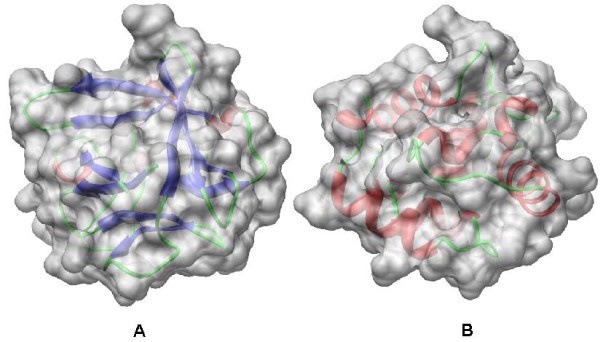
**Proteins with similar shape**. The figure shows two proteins with similar shape but completely different secondary structures. The left protein (PDB: 1BARA) is a *β *class protein and the right protein (PDB: 1RROA) is a *α *class one. The LD descriptor will give them close measurements owing to similar shape of these two proteins.

**Figure 8:**
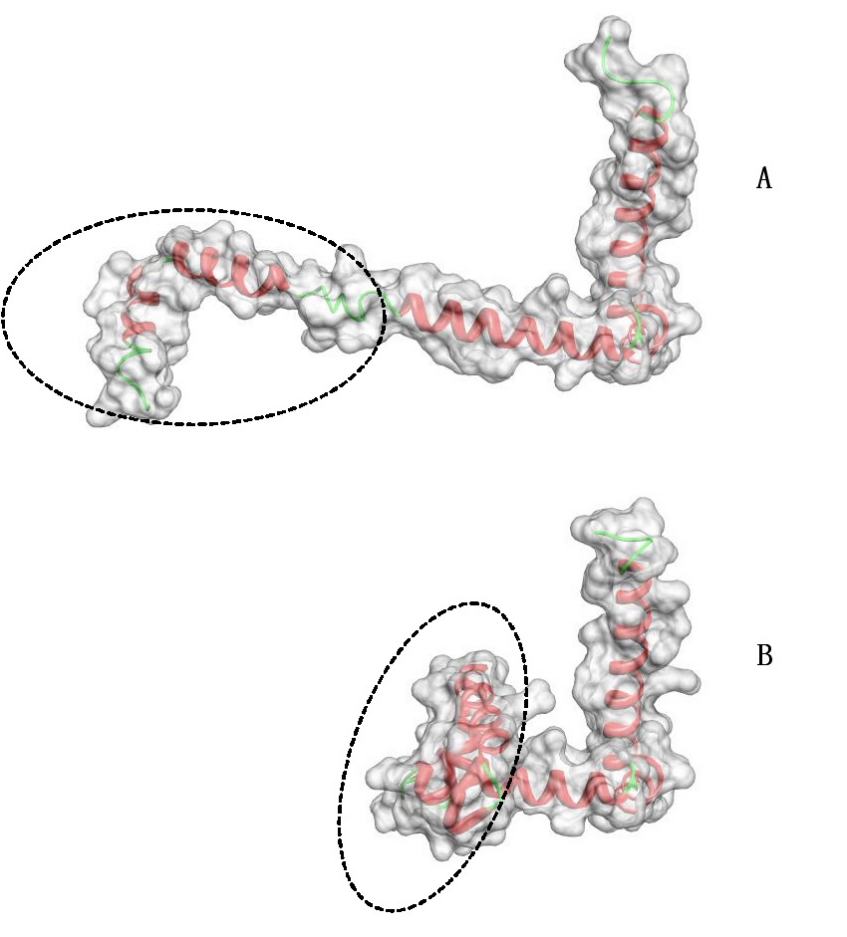
**Proteins with large deformation**. The figure shows two proteins with PDB, 1MI7(top) and 2WRP(bottom). The protein in (A) is transformed into the one in (B) by twisting the long chain (marked by the ellipse) together. The surface regions of the protein in (B) (marked by the ellipse) touch each other, which makes the shape of the protein in (A) deviate a lot from the protein in (B). In this case, the LD descriptor is not able to make a consistent measurement of those two proteins.

We would like to discuss two major limitations of the LD descriptor. First, the protein shape comparison sometimes would lead to the incorrect biological analysis result owing to the inherent properties of shape based approaches. Figure 7 shows an example of two proteins that have a closely similar shape surface but have completely different secondary structure elements. The shape based method would fail to refer to the significant relationship between those two proteins. Second, the LD descriptor is sensitive to large topology change. If the surface regions heavily touch together due to protein deformation, the LD descriptor will fail to give a consistent measurement for that kind of deformation. Figure 8 indicates an example of a case of failure. The long straight chain in the protein (Figure 8(A)) twists together and a large portion surface from different regions touch each other during the deformation process as shown in Figure 8(B). However, in a practical implementation, the LD descriptor could work as a rapid primary filter to obtain the initial results and followed with an option to combine sequence or structure alignment based methods to refine the initial results.

## Conclusion

Capturing the molecule flexibility is important for understanding the properties of proteins. This paper shows how to represent the protein molecular shape by a deformation invariant shape descriptor and demonstrate its efficiency to search against a protein motion database. This descriptor is robust in recognizing the protein conformations since it has a number of attractive properties, namely, insensitivity to topology changes of protein shape, articulated deformation, and skeleton based movement. The experimental results show that the LD descriptor achieved a good performance in the retrieval of a protein motion database. The LD descriptor failed to recognize the large topological changes such as a large contact of the flexible portion with the stable domain and the deformation of the big elongation of the hinge region. Our work contributes to providing an alternative way for protein similarity measures. The proposed shape descriptor is a fast and efficient technique for comparing protein shape, and will be useful for potential applications, such as the drug discovery and structure retrieval.

## Authors' contributions

YIF generated the original idea, executed the research, and wrote the paper. All authors read and approved the final manuscript.
